# Impact of chronic obstructive pulmonary disease and frailty on long-term outcomes and quality of life after transcatheter aortic valve implantation

**DOI:** 10.1007/s40520-017-0864-y

**Published:** 2017-11-28

**Authors:** Artur Dziewierz, Tomasz Tokarek, Pawel Kleczynski, Danuta Sorysz, Maciej Bagienski, Lukasz Rzeszutko, Dariusz Dudek

**Affiliations:** 1 Second Department of Cardiology, Institute of Cardiology, Jagiellonian University Medical College, 17 Kopernika St., 31-501 Krakow, Poland; 2 Department of Interventional Cardiology, Institute of Cardiology, Jagiellonian University Medical College, 17 Kopernika St., 31-501 Krakow, Poland

**Keywords:** Transcatheter aortic valve replacement, Frailty, Aortic valve disease, Elderly

## Abstract

**Background:**

Association between chronic obstructive pulmonary disease (COPD) and long-term mortality as well as the quality of life (QoL) in patients with severe aortic stenosis (AS) undergoing transcatheter aortic valve implantation (TAVI) is still unclear.

**Aim:**

We sought to evaluate the impact of COPD on mortality and QoL of patients with AS undergoing TAVI.

**Methods:**

A total of 148 consecutive patients who underwent TAVI were enrolled and stratified by history of COPD.

**Results:**

Of 148 patients enrolled, 19 (12.8%) patients had a history of COPD. Patients with COPD were high-risk patients with higher prevalence of incomplete revascularization and frailty features. At follow-up of 15.8 months, all-cause mortality in patients with COPD was over four times higher than in patients without COPD [17.8% vs. 52.6%; *p* = 0.002—age/gender-adjusted OR (95% CI) 4.73 (1.69–13.24)]. On the other hand, in Cox regression model, the only independent predictors of all-cause death at long-term follow-up were: incomplete coronary revascularization [HR (95% CI) 5.45 (2.38–12.52); *p* = 0.001], estimated glomerular filtration rate [per 1 ml/min/1.73 m^2^ increase: 0.96 (0.94–0.98); *p* = 0.001], and previous stroke/transient ischemic attack [2.86 (1.17–7.00); *p* = 0.021]. Also, the difference in mortality between patients with and without COPD was not significant after adjustment for the most of frailty indices. Importantly, groups were comparable in terms of QoL at baseline and 12 months.

**Conclusion:**

COPD may pose an important factor affecting long-term outcomes of patients with severe AS undergoing TAVI. However, its effects might be partially related to coexisting comorbidities and frailty.

## Introduction

Concomitant chronic obstructive pulmonary disease (COPD) in patients with severe aortic stenosis (AS) is associated with higher morbidity and mortality after surgical aortic valve replacement (SAVR) [1, 2]. Furthermore, COPD is considered as a marker of poor prognosis and is incorporated in the contemporary cardiac surgery risk prediction models [3, 4]. This results in many elderly patients with severe AS and concomitant COPD being denied surgery and scheduled for transcatheter aortic valve implantation (TAVI) as a lower risk alternative. However, in spite of an improvement in outcomes and quality of life (QoL) after TAVI [5–12], recently, some studies reported a detrimental effect of COPD on outcomes of patients with severe AS undergoing TAVI [13–15]. Nevertheless, the impact of COPD on TAVI results seems to be still unclear [16, 17]. Also, data on the possible associations between COPD, frailty, QoL, and outcomes for patients undergoing TAVI are scarce. Thus, we sought to evaluate the impact of COPD on long-term outcomes and QoL in patients with severe AS undergoing TAVI.

## Methods

A total of 148 consecutive patients with symptomatic severe AS who underwent TAVI were included. All the patients were considered inoperable or high-risk for conventional SAVR [8]. Baseline characteristics and procedural data were collected prospectively. Frailty indices were assessed before TAVI with the Katz index of Independence of Activities in Daily Living, elderly mobility scale (EMS) score, Canadian Study of Health and Aging (CSHA) scale, 5-meter walking test, dominant hand grip strength, and Identification of Seniors at Risk scale [18]. Hand grip strength assessment was performed by 1 physician and it was subjectively distinguished into the following: weak, mild and strong. It was performed using patient’s dominant hand and patients were asked to squeeze dominant hand as tightly as possible (repeated twice) [18]. TAVI procedures were performed using Edwards Sapien, Edwards Sapien XT, Edwards Sapien 3 (Edwards Lifesciences), Medtronic CoreValve/Evolut R (Medtronic, Inc), JenaValve (JenaValve Technology), Lotus (Boston Scientific) and NVT (New Valve Technology). The decision on type and size of the valve as well as access route was at the discretion of the Heart Team and treating physicians. Procedures were performed under general or local anesthesia with mild sedation. Clinical endpoints of the study included all-cause mortality at 30 days and every 6 months up to maximal available follow-up and complications rate up to 12 months. Outcome end points were in accordance with Valve Academic Research Consortium definitions (VARC-2) [19]. QoL was assessed with the self-reported Polish validated version of the EQ-5D-3L questionnaire at baseline and at 12 months after TAVI. The EQ-5D-3L is a health-related QoL measure, consisting of 5 three-level items, representing various aspects of health: mobility, self-care, usual activities, pain/discomfort and anxiety/depression. Respondents can score each domain from one (no problems) to three (extreme problems). The visual analog scale (VAS) score, which is a part of the EQ-5D-3L, was also assessed. For the analysis, patients were stratified by history of COPD as patients with and without concomitant COPD. The diagnosis of COPD was based on the medical history and the results of pulmonary function tests [20]. Spirometry was an obligatory part of the diagnostic work-up for all patients scheduled for TAVI. Standard reference values were used.

Results are presented as number of patients (percentage) or median (interquartile range [IQR]) where applicable. Continuous variables were compared with Mann–Whitney *U* test. Categorical variables were compared with the Chi square test or Fisher’s exact test. Changes in the proportions of patients who reported either “no problems” or “some problems”/“extreme problems” on the EQ-5D-3L between baseline and follow-up visits were analyzed using McNemar’s test. Differences in the VAS score between baseline and follow-up assessments were analyzed with a Wilcoxon signed-rank test. All comparisons between baseline and 12-month measurements were performed excluding unpaired results. Survival curves were constructed using Kaplan–Meier estimates and compared using the log-rank test. Differences in outcomes are presented as adjusted for age/gender odds ratios (OR) with confidence intervals (95% CI). In addition, a multivariable Cox proportional hazards model including baseline variables (except for frailty indices) was used to identify predictors of all-cause mortality at maximal follow-up. Forward selection with a probability value for covariates to enter the model was set at the 0.05 level. Then, additional Cox regression models adjusted for age/gender were constructed to assess associations between COPD, frailty and maximal follow-up all-cause mortality. Results are presented as hazard ratios (HR) with associated 95% CI. A 2-sided *p* value < 0.05 was considered statistically significant. All analyses were performed using SPSS 15.0 (SPSS, Inc, Chicago, IL, USA).

## Results

A total of 148 consecutive patients who underwent TAVI were enrolled. Of them, 19 (12.8%) patients had a history of COPD. Patients with COPD were high-risk individuals with higher prevalence of diabetes mellitus, previous myocardial infarction (MI) and percutaneous coronary intervention as compared to patients without COPD (Table 1). In addition, patients with COPD were more likely to have incomplete coronary revascularization before TAVI. The prevalence of frailty features as assessed with EMS, CSHA, and Katz indices was numerically higher in patients with than without COPD. The grip strength in patients with COPD was lower than in patients without COPD. However, no difference in the prevalence of ‘weak’ category between groups was observed (Table 2). Despite some dissimilarities in baseline risk profile, there were no differences regarding procedural technique and echocardiographic outcomes of TAVI between patients with and without COPD (Table 3).

**Table 1:** Baseline characteristics and echocardiographic data

Variable	All patients (*n* = 148)	Chronic obstructive pulmonary disease		*p* value
		Absent (*n* = 129)	Present (*n* = 19)	
Age, median (IQR) [years]	82.0 (77.0–85.0)	82.0 (78.0–85.0)	78.0 (75.5–83.5)	0.26
Age ≥ 80 years	92 (62.2%)	83 (64.3%)	9 (47.4%)	0.15
Men	56 (37.8%)	46 (35.7%)	10 (52.6%)	0.15
Body mass index, median (IQR) [kg/m^2^]	27.2 (25.2–30.6)	27.3 (25.3–30.2)	28.1 (25.8–33.8)	0.39
Estimated glomerular filtration rate, median (IQR) [ml/min/1.73 m^2^]	56.5 (40.0–72.0)	54.0 (39.0–72.0)	66.0 (45.0–80.0)	0.19
New York Heart Association class				0.24
I	0 (0.0%)	0 (0.0%)	0 (0.0%)	
II	41 (27.7%)	36 (27.9%)	5 (26.3%)	
III	97 (65.5%)	86 (66.7%)	11 (57.9%)	
IV	10 (6.8%)	7 (5.4%)	3 (15.8%)	
Arterial hypertension	139 (93.9%)	120 (93.0%)	19 (100.0%)	0.61
Diabetes mellitus	48 (32.4%)	38 (29.5%)	10 (52.6%)	0.044
Atrial fibrillation	52 (35.1%)	44 (34.1%)	8 (42.1%)	0.50
Previous myocardial infarction	48 (32.4%)	38 (29.5%)	10 (52.6%)	0.044
Previous percutaneous coronary intervention	43 (29.1%)	33 (25.6%)	10 (52.6%)	0.015
Previous coronary artery bypass grafting	28 (18.9%)	26 (20.2%)	2 (10.5%)	0.53
Chronic total occlusion	14 (9.5%)	8 (6.2%)	6 (31.6%)	0.003
Incomplete revascularization	22 (14.9%)	15 (11.6%)	7 (36.8%)	0.010
Previous stroke/transient ischemic attack	17 (11.5%)	13 (10.1%)	4 (21.1%)	0.24
Pacemaker	17 (11.5%)	17 (13.2%)	0 (0.0%)	0.13
Logistic Euroscore I, median (IQR) [%]	14.5 (10.0–22.7)	14.0 (10.0–22.0)	15.0 (13.4–23.5)	0.10
The Society of Thoracic Surgeons score, median (IQR) [%]	6.2 (4.0-17.3)	7.0 (4.0–17.0)	5.5 (3.8–30.0)	0.65
Maximal transvalvular gradient, median (IQR) [mmHg]	86.0 (69.0–103.0)	87.0 (70.0–104.0)	80.0 (71.0–90.5)	0.21
Mean transvalvular gradient, median (IQR) [mmHg]	50.0 (42.0–63.0)	50.0 (42.0–64.5)	47.0 (42.5–53.0)	0.37
Aortic valve area, median (IQR) [cm^2^]	0.7 (0.6–0.8)	0.7 (0.5–0.8)	0.7 (0.6–1.0)	0.09
Left ventricle ejection fraction, median (IQR) [%]	60.0 (50.0–65.0)	60.0 (50.0–65.0)	60.0 (49.5–65.0)	0.57
Aortic regurgitation				0.034
0	48 (32.4%)	46 (35.7%)	2 (10.5%)	
1	75 (50.7%)	62 (48.1%)	13 (68.4%)	
2	20 (13.5%)	18 (14.0%)	2 (10.5%)	
3	5 (3.4%)	3 (2.3%)	2 (10.5%)	
Systolic pulmonary artery pressure ≥ 46 mmHg	65 (43.9%)	54 (41.9%)	11 (57.9%)	0.19

**Table 2:** Frailty indices in patients with and without chronic obstructive pulmonary disease

Variable	Categories	All patients (*n* = 148)	Chronic obstructive pulmonary disease		*p* value
			Absent (*n* = 129)	Present (*n* = 19)	
5-meter walking test (seconds)	≥ 6, frail	21 (14.2%)	17 (13.2%)	4 (21.1%)	0.48
Elderly mobility scale (points)	< 10, frail	8 (5.4%)	5 (3.9%)	3 (15.8%)	0.11
	10–13	93 (62.8%)	83 (64.3%)	10 (52.6%)	
	> 13	47 (31.8%)	41 (31.8%)	6 (31.6%)	
Canadian Study of Health and Aging scale (points)	1–3	87 (58.8%)	78 (60.5%)	9 (47.4%)	0.06
	4	44 (29.7%)	37 (28.7%)	7 (36.8%)	
	5, frail	3 (2.0%)	1 (0.8%)	2 (10.5%)	
	6–7, frail	14 (9.5%)	13 (10.1%)	1 (5.3%)	
Katz index (points)	< 6, frail	19 (12.8%)	14 (10.9%)	5 (26.3%)	0.07
Grip strength (grade)	Weak, frail	7 (4.7%)	6 (4.7%)	1 (5.3%)	0.025
	Mild	14 (9.5%)	9 (7.0%)	5 (26.3%)	
	Strong	127 (85.8%)	114 (88.4%)	13 (68.4%)	
Identification of Seniors at Risk scale (points)	≥ 2, functional decline, frail	53 (35.8%)	46 (35.7%)	7 (36.8%)	0.92

**Table 3:** Procedural and echocardiographic data after the procedure

Variable	All patients (*n* = 148)	Chronic obstructive pulmonary disease		*p* value
		Absent (*n* = 129)	Present (*n* = 19)	
Access type				0.84
Transfemoral	117 (79.1%)	102 (79.1%)	15 (78.9%)	
Transapical	28 (18.9%)	24 (18.6%)	4 (21.1%)	
Transaortic	2 (1.4%)	2 (1.6%)	0 (0.0%)	
Subclavian	1 (0.7%)	1 (0.8%)	0 (0.0%)	
Device implanted				0.23
Corevalve / Evolut R	29 (19.6%)	28 (21.7%)	1 (5.3%)	
Edwards Sapien	95 (64.2%)	83 (64.3%)	12 (63.2%)	
Jena	10 (6.8%)	7 (5.4%)	3 (15.8%)	
Lotus	9 (6.1%)	7 (5.4%)	2 (10.5%)	
NVT	5 (3.4%)	4 (3.1%)	1 (5.3%)	
Prosthesis size				0.35
23 mm	30 (20.3%)	26 (20.2%)	4 (21.1%)	
25 mm	8 (5.4%)	7 (5.4%)	1 (5.3%)	
26 mm	56 (37.8%)	52 (40.3%)	4 (21.1%)	
27 mm	8 (5.4%)	6 (4.7%)	2 (10.5%)	
29 mm	38 (25.7%)	32 (24.8%)	6 (31.6%)	
31 mm	8 (5.4%)	6 (4.7%)	2 (10.5%)	
Prosthesis size, median (IQR) [mm]	26.0 (25.0–29.0)	26.0 (25.0–29.0)	27.0 (25.5–29.0)	0.33
Maximal transvalvular gradient, median (IQR) [mmHg]	13.0 (10.0–19.0)	13.0 (10.0–19.0)	16.0 (13.0–21.0)	0.13
Median transvalvular gradient, median (IQR) [mmHg]	7.4 (5.1–10.0)	7.0 (5.0–10.0)	9.0 (6.0–11.0)	0.12
Left ventricle ejection fraction, median (IQR) [%]	48.0 (41.0–55.0)	49.0 (43.0–55.0)	45.0 (32.5–60.0)	0.61
Aortic regurgitation				0.60
0	84 (56.8%)	71 (55.0%)	13 (68.4%)	
1	55 (37.2%)	50 (38.8%)	5 (26.3%)	
2	7 (4.7%)	6 (4.7%)	1 (5.3%)	
3	2 (1.4%)	2 (1.6%)	0 (0.0%)	
Radiation dose, median (IQR) [mGy]	721.0 (632.5–827.5)	713.0 (632.0–823.0)	789.0 (639.5–905.5)	0.13
Contrast medium load, median (IQR) [ml]	75.0 (50.0–137.5)	75.0 (50.0–100.0)	75.0 (50.0–125.0)	0.80
Fluoroscopy time, median (IQR) [min]	13.0 (12.0–15.0)	13.0 (12.0–15.0)	13.0 (12.0–14.0)	0.70

**Fig. 1:**
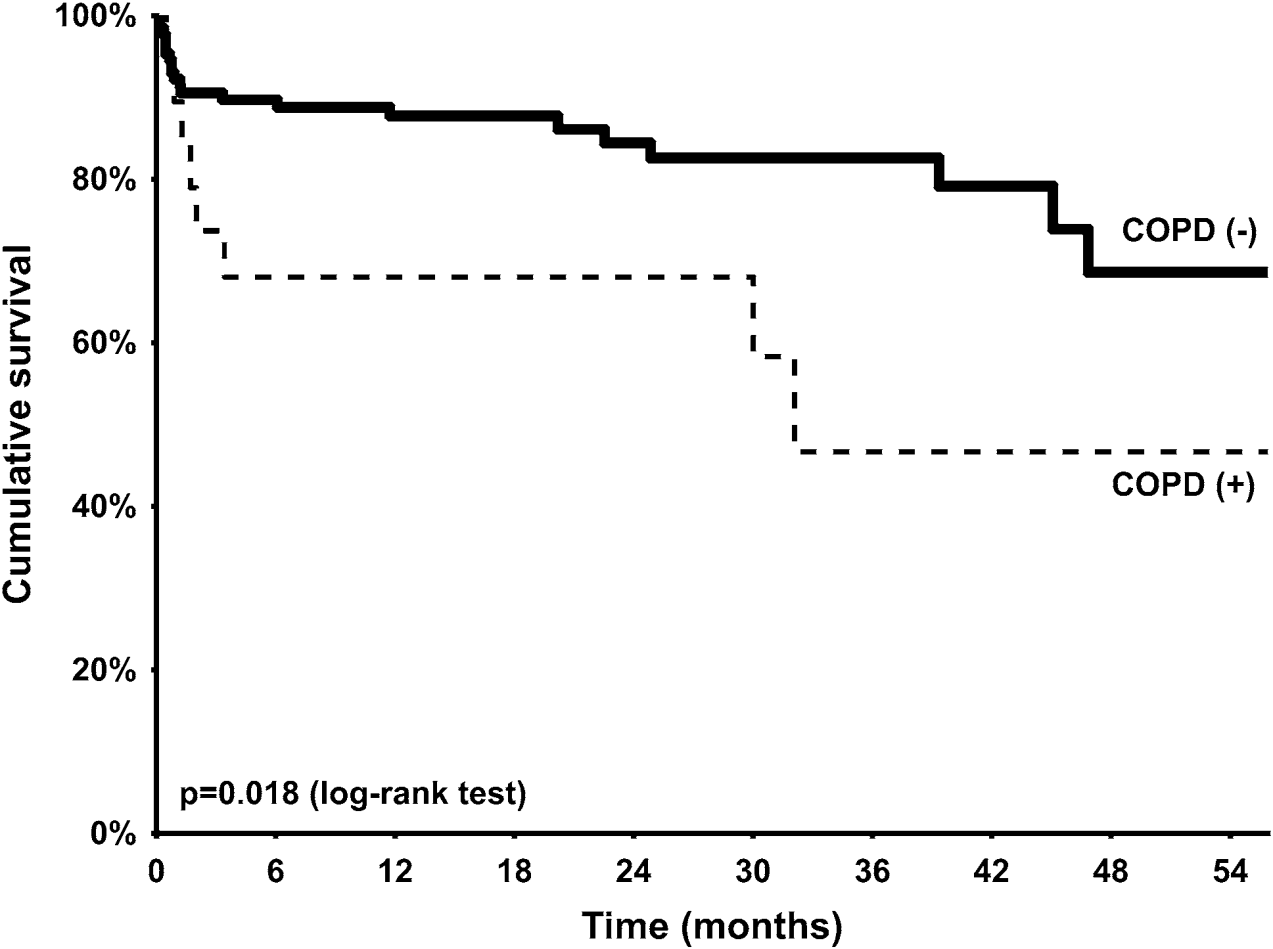
Kaplan–Meier curves for survival after transcatheter valve implantation stratified by chronic obstructive pulmonary disease (COPD) status (without *COPD* solid line; with *COPD* dotted line)

The median length of hospital stay was comparable between groups [COPD (−) vs. COPD (+): 10.0 (8.0–13.0) vs. 9.5 (7.0–12.0) days; *p* = 0.50]. No difference in the rate of bleeding [32.6% vs. 36.8%; *p* = 0.71, age/gender-adjusted OR (95% CI) 1.23 (0.44–3.39)] as well as blood transfusion [29.5% vs. 31.6%; *p* = 0.85—age/gender-adjusted OR (95% CI) 1.12 (0.39–3.23)] during hospital stay was observed. Similarly, the rate of grade 3 acute kidney injury (AKI) was comparable between groups [5.4% vs. 5.4%; *p* = 1.00—age/gender-adjusted OR (95% CI) 0.87 (0.10–7.96)]. At 30 days, no difference in all-cause mortality was observed [7.8% vs. 10.5%; *p* = 0.65—age/gender-adjusted OR (95% CI) 1.37 (0.27–6.90)]. However, at 12 months all-cause mortality in patients with COPD was over 4 times higher than in patients without COPD [11.6% vs. 36.8%; *p* = 0.010—age/gender-adjusted OR (95% CI) 4.19 (1.34–13.09)]. This difference in mortality was maintained during the median follow-up of 15.8 (6.4–33.1) months [17.8% vs. 52.6%; *p* = 0.002—age/gender-adjusted OR (95% CI) 4.73 (1.69–13.24)]—Fig. 1. On the other hand, the difference in mortality between patients with and without COPD was not significant after adjustment for most of the frailty indices (Table 4). In addition, in Cox regression model, COPD was not identified as an independent predictor of long-term all-cause mortality. The only independent predictors were: incomplete coronary revascularization [HR (95% CI) 5.45 (2.38–12.52); *p* = 0.001], estimated glomerular filtration rate [HR (95% CI) per 1 ml/min/1.73 m^2^ increase: 0.96 (0.94–0.98); *p* = 0.001], and previous stroke/transient ischemic attack [HR (95% CI) 2.86 (1.17–7.00); *p* = 0.021]. In addition, a trend towards higher risk of MI [1.6% vs. 10.5%; *p* = 0.08—age/gender-adjusted OR (95% CI) 8.52 (0.94–77.55)], cerebrovascular accidents [5.4% vs. 15.8%; *p* = 0.12—age/gender-adjusted OR (95% CI) 2.77 (0.63–12.28)], and new onset of atrial fibrillation [5.4% vs. 15.8%; *p* = 0.012—age/gender-adjusted OR (95% CI) 2.45 (0.53–11.32)] at 12 months was noted in patients with COPD. New permanent pacemaker implantation was required in 17.1% of patients without COPD and 10.5% of patients with COPD [*p* = 0.74—age/gender-adjusted OR (95% CI) 0.60 (0.13–2.80)].

**Table 4:** Impact of chronic obstructive pulmonary disease and frailty indices on maximal follow-up all-cause mortality

Variable	Age/gender-adjusted HR (95% CI)	*p* value
5-meter walking test ≥ 6 s	14.71 (6.50–33.3)	< 0.001
Chronic obstructive pulmonary disease	1.47 (0.66–3.26)	0.34
Elderly mobility scale < 10 points	13.80 (5.34–35.64)	< 0.001
Chronic obstructive pulmonary disease	1.41 (0.58–3.42)	0.45
Canadian Study of Health and Aging scale 5–7 points	39.10 (15.85–96.46)	< 0.001
Chronic obstructive pulmonary disease	1.82 (0.79–4.18)	0.16
Katz index < 6 points	13.92 (6.29–30.79)	< 0.001
Chronic obstructive pulmonary disease	1.10 (0.48–2.56)	0.82
Weak grip strength	28.84 (10.54–78.87)	< 0.001
Chronic obstructive pulmonary disease	3.09 (1.37–6.95)	0.006
Identification of Seniors at Risk scale ≥ 2 points	5.25 (2.20–12.55)	< 0.001
Chronic obstructive pulmonary disease	2.57 (1.16–5.70)	0.021

**Fig. 2:**
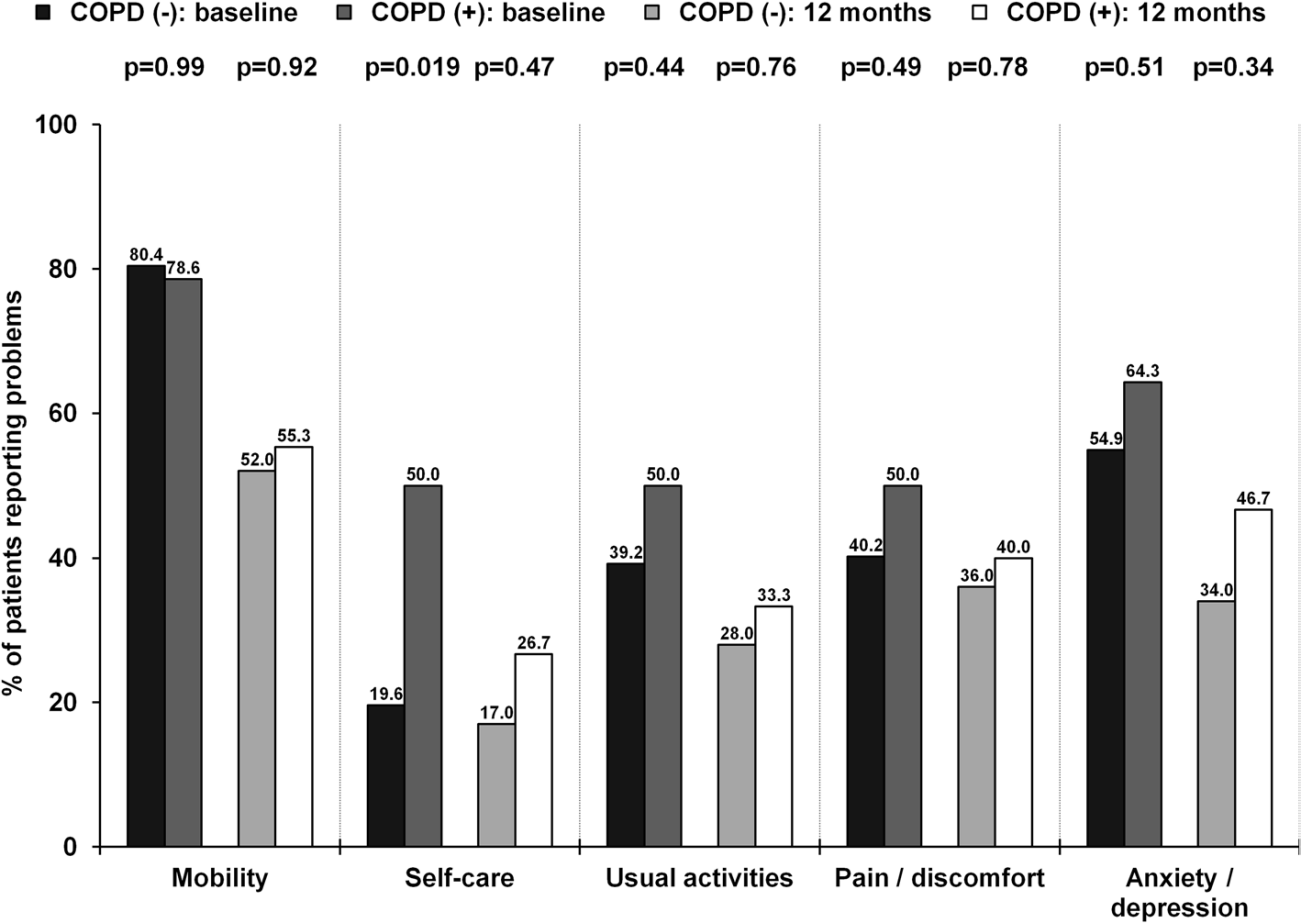
Proportions of patients that report either “some problems”/“extreme problems” for each category of the EQ-5D-3L at baseline and 12 months

In general, baseline QoL parameters as assessed with the EQ-5D-3L were comparable between groups. However, a higher rate of reporting either “some problems”/“extreme problems” in terms of self-care in patients with COPD as compared with those without COPD was observed (Fig. 2). An improvement in QoL after 12 months was confirmed for both groups for mobility and anxiety/depression components of the EQ-5D-3L. On the contrary, an improvement in self-care was confirmed only for patients with COPD. The median VAS score at baseline [40.0 (30.0–50.0) vs. 45.0 (25.0–60.0); *p* = 0.83] and 12 months [70.0 (60.0–80.0) vs. 70.0 (60.0–77.5); *p* = 0.56] was comparable between groups. No difference in change of VAS from baseline to 12 months was found [25.0 (15.0–40.0) vs. 25.0 (10.0–30.0); *p* = 0.58].

## Discussion

The major finding of our study is that COPD had a detrimental effect on long-term all-cause mortality in patients with severe AS scheduled for TAVI. These results were maintained after the adjustment for age and gender, while no difference in mortality between patients with and without COPD was observed after adjustment for most of the frailty indices. Furthermore, COPD was not identified as an independent predictor of long-term all-cause mortality. Thus, effects of COPD might be partially related to coexisting comorbidities and frailty. Importantly, our findings stay in line with the previous studies reporting association between COPD and adverse outcomes after TAVI [13–15, 21, 22]. However, other studies presented contradictory results suggesting no influence of COPD on mortality after TAVI [16, 17]. For instance, in multicenter Canadian experience, the strongest predictor of late mortality was COPD followed by chronic kidney disease, chronic atrial fibrillation, and frailty [21]. A recent large meta-analysis [13] has reported negative impact of COPD on both short- and long-term all-cause mortality (30 days OR 1.43, 95% CI 1.14–1.79; >2 years: HR 1.34, 95% CI 1.12–1.61). COPD was also associated with increased short- and mid-term cardiovascular mortality (30 days OR 1.29, 95% CI 1.02–1.64; 1 year: HR 1.09, 95% CI 1.02–1.17). Furthermore, patients with COPD had 2 times higher risk for AKI than patients without COPD. It has been suggested that COPD is likely to cause a reduction of renal blood flow [23], and that periprocedural impairment of heart function and the occurrence of hypoxemia and hypercapnia can lead to deterioration in renal function [13, 24]. In addition, COPD has been reported as an important predictor of stroke after TAVI [25]. In our study, only a trend towards higher risk of MI, new onset atrial fibrillation, and cerebrovascular accidents was noted in patients with COPD at 12-month follow-up. Numerically higher rates of those adverse events may suggest that those results could reach statistical significance with a large sample. Importantly, paroxysmal atrial fibrillation is quite common among patients with COPD and might be related to COPD exacerbation and treatment with theophylline [26, 27]. Also, COPD and coronary artery disease share several risk factors such as smoking habit and aging. Thus, it is expected that a considerable number of patients with COPD scheduled for TAVI also have concomitant coronary artery disease. In our study, these patients were more likely to have chronic total occlusions and incomplete revascularization of coronary arteries before TAVI. This result might be related to a higher prevalence of diabetes mellitus, previous MI and percutaneous coronary intervention in the COPD group. On the other hand, a link between COPD and systemic inflammatory response was suggested. That way COPD may hasten the progression of atherosclerotic disease as well as the destabilization of existing atherosclerotic plaques [27]. Interestingly, a systemic inflammatory response measured by elevation of C-reactive protein, interleukin-6, and interleukin-8 levels has been shown to be associated with worse prognosis in patients undergoing TAVI [28].

Frailty and sarcopenia have been shown to be a frequent condition in elderly patients and should be considered when dealing with invasive care in the elderly [18, 29]. Importantly, both frailty and sarcopenia are common among patients with a chronic respiratory disease [30]. Also, those were shown to be associated with worse outcomes of patients with severe AS undergoing TAVI [18, 29]. In our study, the prevalence of frailty was numerically higher in patients with than without COPD and the presence of frailty was strongly associated with long-term all-cause mortality. On the other hand, the observed difference in mortality between patients with and without COPD was not significant after correction for frailty indices. However, in patients with COPD and severe AS, it may be difficult to distinguish the precise contribution of each pathology, when progressive dyspnea appears [31]. Also, in inoperable patients with severe AS and COPD, it seems crucial to define the border between utility and futility, when the condition is too far advanced and the risk too high even for this less invasive procedure.

Evaluation of QoL seems to be an important index as frequently not a reduction in mortality but improvement in daily life comfort is most desirable by patients themselves [9–11]. Importantly, improvement in QoL after TAVI may be higher than observed after SAVR, even with the use of minimally-invasive surgical techniques (mini-thoracotomy, mini-sternotomy) [11]. However, these benefits in terms of improvement in QoL after TAVI may be affected by the presence of comorbidities, including COPD. De Oliveira et al. [32] suggested that QoL in patients with COPD was lower than expected. Also, up to 20% of patients with COPD were on antidepressants, which had a possible influence on QoL outcomes [32]. Furthermore, increasing level of severity of COPD was associated with a significant decline in the EQ-5D VAS scores and utility scores [33]. In our study, an improvement in QoL at 12 months after TAVI was confirmed for patients both with and without COPD for mobility and anxiety/depression components of the EQ-5D-3L. On the contrary, an improvement in self-care was confirmed only for patients with COPD. No difference in VAS change during follow-up between groups was reported. This might suggest an equal response to TAVI in terms of QoL regardless of COPD status.

Several important limitations of the present study should be acknowledged. First, the study has all the limitations of a single-center registry. Second, patients were not stratified by the severity of COPD. It is probable that the relative impact of COPD on outcomes is dependent on disease severity. Third, symptoms control and a history of exacerbations, as well as type of treatment, were not assessed. Finally, the tools used for QoL and frailty assessment were non-disease-specific instruments. Prevalence of depression and the use of antidepressants in patients with COPD were not evaluated. However, despite several limitations, our study represents a complete analysis of consecutive patients without any exclusion criteria and with follow-up data available for all patients.
